# Metagenomic Insights Into Functional and Taxonomic Compositions of an Activated Sludge Microbial Community Treating Leachate of a Completed Landfill: A Pathway-Based Analysis

**DOI:** 10.3389/fmicb.2021.640848

**Published:** 2021-04-30

**Authors:** Shohei Yasuda, Toshikazu Suenaga, Laura Orschler, Shelesh Agrawal, Susanne Lackner, Akihiko Terada

**Affiliations:** ^1^Department of Chemical Engineering, Tokyo University of Agriculture and Technology, Koganei, Japan; ^2^Global Innovation Research Institute, Tokyo University of Agriculture and Technology, Fuchu, Japan; ^3^Department of Civil and Environmental Engineering Science, Institute IWAR, Chair of Wastewater Engineering, Technical University of Darmstadt, Darmstadt, Germany

**Keywords:** landfill, leachate, activated sludge, metagenome, *Methyloceanibacter*, metagenomic screening

## Abstract

Upcycling wastes into valuable products by mixed microbial communities has recently received considerable attention. Sustainable production of high-value substances from one-carbon (C1) compounds, e.g., methanol supplemented as an external electron donor in bioreactors for wastewater treatment, is a promising application of upcycling. This study undertook a gene-centric approach to screen valuable production potentials from mixed culture biomass, removing organic carbon and nitrogen from landfill leachate. To this end, the microbial community of the activated sludge from a landfill leachate treatment plant and its metabolic potential for the production of seven valuable products were investigated. The DNA extracted from the activated sludge was subjected to shotgun metagenome sequencing to analyze the microbial taxonomy and functions associated with producing the seven products. The functional analysis confirmed that the activated sludge could produce six of the valuable products, ectoine, polyhydroxybutyrate (PHB), zeaxanthin, astaxanthin, acetoin, and 2,3-butanediol. Quantification of the detected functional gene hit numbers for these valuable products as a primary trial identified a potential rate-limiting metabolic pathway, e.g., conversion of *L*-2,4-diaminobutyrate into *N*-γ-acetyl-L2,4,-diaminobutyrate during the ectoine biosynthesis. Overall, this study demonstrated that primary screening by the proposed gene-centric approach can be used to evaluate the potential for the production of valuable products using mixed culture or single microbe in engineered systems. The proposed approach can be expanded to sites where water purification is highly required, but resource recovery, or upcycling has not been implemented.

## Introduction

Microbial resource management (MRM), which enables a better understanding and harnessing of microorganisms in ecosystems for sustainable development, has been advocated and implemented in engineered systems (Verstraete, 2007). One application of MRM is recovery or upcycling of valuable resources from wastes and wastewater (McCarty et al., 2011). MRM does not hinge upon the use of one species, but rather a myriad of uncultured microorganisms with various functions that are not fully understood. Accordingly, exploring and comprehending microbial community compositions and functions could pave the way for the innovation of novel bioprocesses, contributing to resource recovery, production of valuable materials, and energy-saving by wastewater treatment systems.

Among targeted sites amenable to MRM applications, bioreactors treating industrial water and wastewater, e.g., waste produced during food processing and by livestock industries (Matassa et al., 2015; Spiller et al., 2020) and landfill leachate, are promising. From a societal perspective, the demand for appropriate leachate treatment is expected to surge as the amount of waste increases from 2.0 billion tons in 2016 to an expected 3.4 billion tons by 2050 (Kaza et al., 2018). Landfill leachate consists of small molecular organic substances and biodegradable volatile fatty acids in the early stages of the landfill process, while ammonia and persistent organic substances are the main components in the later stages following microbial decomposition of major organic waste constituents (Costa et al., 2019). Methanol is often used as a C1 electron donor to replenish insufficient supplies of electron donors for nitrogen oxides produced during ammonia oxidation in landfill leachate treatment after long-term implementation to facilitate denitrification (Ilies and Mavinic, 2001). Recent studies have shown that methanotrophs and methylotrophs, which are both C1-assimilating bacteria, synthesize valuable products including biodegradable polymers and active ingredients in cosmetics (Zamakhaeva et al., 2017). Given that leachate treatment plants likely harbor microbial consortia that assimilate C1 compounds and ammonia, leachate can be expected to function as a site at which these simple wastewater constituents can be converted into valuable products.

Methanol supplied to activated sludge of a wastewater treatment plant and indigenously high salinity (1–3%) triggers changes in microbial community compositions (Osaka et al., 2006). Moreover, microbial communities enriched by high methanol concentrations are reportedly capable of producing high-value products such as ectoine (Doronina et al., 2010), polyhydroxybutyrate (PHB; Zamakhaeva et al., 2017), acetoin (3-hydroxy-2-butanone; Schendel et al., 1990; Gogleva et al., 2010), and carotenoids (Berry et al., 2003; Lee et al., 2004; Guo et al., 2017). Despite their benefits (Supplementary Table 1), chemical industries face challenges with cost-effective and high-yield production of these products (Pastor et al., 2010; Xiao and Lu, 2014; Levett et al., 2016; Zhang, 2018). However, the development of a biorefinery platform based on the MRM concept has the potential to overcome the challenges. Therefore, bioprocesses based on mixed microbial communities are potential surrogates to commercial chemical processes. Accordingly, it is important to explore and harness effective microbes harboring the genetic potential to synthesize valuable products. However, the phylogeny and physiology of such microbes and varieties of targeted valuable products remain mostly unexploited.

The design and operation of bioreactors for treating wastewater has been reliant on an empirical top-down approach, in which macro-scale variables, e.g., pH and dissolved oxygen, are precisely controlled because of limited understanding of the intricate and diverse compositions of microbial communities in wastewater (Lawson et al., 2019). However, recent advances in high-throughput sequencing technologies, genome-centric with binning, and multi-omics approaches have enabled analysis of micro-scale microbiological processes, including metabolic networks, and interactions among microbes (Nobu et al., 2015; Lawson et al., 2017). Despite massive and invaluable information on functions and taxonomies of microbial communities, genome-centric and multi-omics approaches, however, are labor-intensive and time-consuming, and require strong expertise on microbial ecology and bioinformatics, which does not allow engineers to fully harness the approaches. Therefore, a simple platform to rapidly explore the possibility for a microbial consortium to produce valuable products is demanded.

This study advocates a simple gene-centric method without binning to screen the capability of synthesizing valuable products. Taking a landfill as an MRM site, the analysis was conducted to elucidate the microbial community composition and functions of activated sludge in a landfill leachate treatment system in which methanol was supplemented as an electron donor for nitrogen removal. Using shotgun metagenomics analysis, the metabolic potential for the production of valuable products was targeted. The specific goals of this study were: (i) to determine if an individual species of bacteria or microbial community in the activated sludge possesses the metabolic potential for the synthesis of targeted products at the gene level; and (ii) to infer potential pathways that could act as rate-limiting steps during production.

## Materials and Methods

### Sampling

Activated sludge of a landfill leachate treatment plant at a completed landfill (Tokyo, Japan) was collected on 20 July 2018. The landfill was in operation for several decades, during which time the leachate was treated by an activated sludge system in a sequencing batch mode. The sequencing batch reactor (SBR) had an effective volume of 740 m^3^ and received 74.2 m^3^/day of leachate on average. One cycle of the SBR operation lasted 2 h and consisted of 10 min for inflow, 45 min for mixing to ensure anoxic conditions, 15 min for aeration, and 50 min for settling and outflow of the treated water. Prior to entry of the influent to the SBR, the leachate was mixed with groundwater at a volumetric ratio of 3 (leachate): 1 (groundwater). Tables 1, 2 summarize the operating conditions, and the leachate characteristics and SBR performance, respectively. The organic carbon concentration was low (BOD < 15 mg/L and COD < 50 mg/L), whereas the ammonium concentration in the leachate was as high as 150 mg-N/L. Because of the imbalance of the carbon/nitrogen ratio, about 5.3 L of 50% v/v methanol was added during each inflow period as an external electron donor for denitrification. The methanol amount allowed a relative loading of COD/T-N (kg-COD/kg-N) of 4.38 ± 1.80, which is higher than the denitrification stoichiometry with methanol.

**TABLE 1:** Treating condition of leachate.

Influent		Effluent	HRT	MLSS	Inf. COD loading	Inf. T-N loading	Methanol-based COD loading	COD/T-N ratio
Leachate	Groundwater	(m^3^/day)	(day)	(mg/L)	(kg-COD/day)	(kg-N/day)	(kg-COD/day)	(kg-COD/kg-N)
(m^3^/day)	(m^3^/day)					(*N* = 18)		(*N* = 18)
74.2 ± 40.0	13.2 ± 11.1	88.6 ± 22.3	4.61 ± 1.06	7286 ± 303	2.92 ± 0.61	9.47 ± 2.54	37.4 ± 13.1	4.38 ± 1.80

*The number of samples (N) was 62 unless otherwise described.*

**TABLE 2:** The SBR performances for leachate treatment.

Constituent	BOD	COD	SS	pH	Transparency	Temperature	NH_4_^+^-N	NO_2_^–^-N	NO_3_^–^-N	T-N	Cl^–^	EC	Salinity
	(mg/L)	(mg/L)	(mg/L)	(-)	(cm)	(°C)	(mg-N/L)	(mg-N/L)	(mg-N/L)	(mg-N/L)	(mg/L)	(μS/cm)	(%)
Leachate	11.8 ± 1.5	43.9 ± 9.2	4.3 ± 11.2	7.65 ± 0.07	97 ± 10	28.3 ± 1.1	151.31 ± 49.53	0.48 ± 0.13	3.34 ± 0.63	158.52 ± 51.19	7693 ± 1767	21,933 ± 4557	1.23 ± 0.27
	(*N* = 9)	(*N* = 62)	(*N* = 62)	(*N* = 62)	(*N* = 62)	(*N* = 62)	(*N* = 18)	(*N* = 18)	(*N* = 18)	(*N* = 18)	(*N* = 3)	(*N* = 3)	(*N* = 3)
Treated water	1.0 ± 0.0	9.8 ± 1.8	1.4 ± 0.5	7.35 ± 0.08	100 ± 0	28.8 ± 1.4	0.049 ± 0.040	0.013 ± 0.024	1.76 ± 1.13	2.98 ± 0.79	4873 ± 454	14,700 ± 1058	0.79 ± 0.07
	(*N* = 9)	(*N* = 62)	(*N* = 62)	(*N* = 62)	(*N* = 62)	(*N* = 62)	(*N* = 18)	(*N* = 18)	(*N* = 18)	(*N* = 18)	(*N* = 3)	(*N* = 3)	(*N* = 3)

### DNA Extraction

Twenty liters of activated sludge were taken from the SBR and immediately transferred to the laboratory. After thoroughly mixing the activated sludge, 15 mL of wastewater containing 22.2 mg-wet weight-activated sludge were used for DNA extraction by a DNA extraction kit (Fast DNA^TM^ Spin Kit for Soil, MP-Biomedicals, Santa Ana, CA, United States) according to the manufacturer’s protocol. The DNA concentration was measured using a spectrophotometer (NanoDrop 2000c, Thermo Scientific, Wilmington, DE, United States).

### Sequence Processing and Metagenomic Assembly

The sequencing and metagenomic assembly have been described in detail in a previous study (Yasuda et al., 2020). Briefly, sequencing was performed with an Ion Torrent (ION Torrent Ion S5) using an Ion 530^TM^ Chip. Torrent Suite v 4.4.2 (Thermo Fisher Scientific, Germany) was used for base calling and to run multiplexing according to the manufacturer’s protocol. After quality filtering of metagenomic reads and trimming both sides of the reads to ensure a Q20 quality score, 20.24 million reads were processed, giving 13,005,150 reads. Megahit v 1.1.3 (Li et al., 2015) was used at the minimum contig length of 1,000 bp for assembly, resulting in acquisition of 104.86 Mbp contigs. Mapping the reads to the assembly was accomplished with Bowtie2 v 2.3.4.2 (Langmead and Salzberg, 2013), and the subsequent identification of single-copy bacterial and archaeal genes was achieved using HMMER v 3.2 (Finn et al., 2011). Taxonomy was assigned to contigs using Centrifuge v 1.0.3 (Kim et al., 2016). The latest taxonomic information was used for classification (Waite et al., 2017, 2020; Parks et al., 2018). Default parameters were used for all software unless otherwise specified.

### Functional Genes and Metabolic Pathways of Valuable Products

Amino-acid sequence data obtained by the HMMER procedure were used to query the Kyoto Encyclopedia of Genes and Genomes (KEGG; Kanehisa and Goto, 2000) with GhostKOALA v 2.1 (Kanehisa et al., 2016). The existence of functional genes and metabolic pathways associated with the production of valuable products by either microbial communities or a single species of indigenous bacteria were confirmed. Based on a literature survey for valuable products microbiologically synthesized from C1 compounds, the metabolic pathways and related functional genes for the production of ectoine (Pastor et al., 2010), PHB (Orita et al., 2014), carotenoids (astaxanthin and zeaxanthin; Ye and Bhatia, 2012; Zhang, 2018), and acetoin (Nielsen et al., 2010) were selected. Carotenoids are synthesized from acetyl-CoA to terpenoid backbone synthesis via either a mevalonate pathway or a non-mevalonate pathway (Supplementary Figure 1 and Supplementary Table 1). The majority of bacteria possess the latter, whereas some use the former pathway (Hoshino and Gaucher, 2018; Rico et al., 2019). Therefore, this study investigated both pathways.

This research assumed that all eubacteria possess central metabolisms, e.g., glycolysis and the TCA cycle. Essential functional genes and enzymes regulating biosynthesis of each valuable product are listed in Table 3. In addition to these compounds that C1-assimilating bacteria are potentially able to synthesize, 2,3-butanediol, which is a promising fuel that is chemically produced from acetoin (Nielsen et al., 2010; Ji et al., 2011), and lutein, which is a carotenoid mainly produced by plants and is known to play a role in vision enhancement (Ye and Bhatia, 2012; Nwachukwu et al., 2016; Zhang, 2018), were also selected.

**TABLE 3:** Functional genes and enzymes related to production of valuable products. The functional genes of the metabolic pathways, consist of terpenoid backbone synthesis and carotenoid biosynthesis, are shown in Supplementary Table 1.

Valuable products	Functional genes	Enzymes	Literature
Ectoine	*lysC asd ectB ectA ectC*	Aspartate kinase [EC:2.7.2.4] Aspartate-semialdehyde dehydrogenase [EC:1.2.1.11] Diaminobutyrate-2-oxoglutarate transaminase [EC:2.6.1.76] L-2,4-diaminobutyric acid acetyltransferase [EC:2.3.1.178] L-ectoine synthase [EC:4.2.1.108]	Pastor et al., 2010
PHB	*phaA phaB phaC*	Acetyl-CoA C-acetyltransferase [EC:2.3.1.9] Acetoacetyl-CoA reductase [EC:1.1.1.36] Polyhydroxyalkanoate synthase subunit PhaC [EC:2.3.1.-]	Orita et al., 2014
Lutein	Terpenoid backbone synthesis-related genes	Mevalonate pathway (MVA)-related enzymes The methylerythritol phosphate pathway (MEP)-related enzymes	Zhang, 2018
	Carotenoid biosynthesis-related genes	Carotenoid biosynthesis-related enzymes	Misawa et al., 1995; Ye and Bhatia, 2012
Astaxanthin	Terpenoid backbone synthesis-related genes	Mevalonate pathway (MVA)-related enzymes The methylerythritol phosphate pathway (MEP)-related enzymes	Zhang, 2018
	Carotenoid biosynthesis-related genes	Carotenoid biosynthesis-related enzymes	Misawa et al., 1995; Ye and Bhatia, 2012
Zeaxanthin	Terpenoid backbone synthesis-related genes	Mevalonate pathway (MVA)-related enzymes The methylerythritol phosphate pathway (MEP)-related enzymes	Zhang, 2018
	Carotenoid biosynthesis-related genes	Carotenoid biosynthesis-related enzymes	Misawa et al., 1995; Ye and Bhatia, 2012
Acetoine	*E2.2.1.6L E2.2.1.6S ilvM alsD BDH butA, budC*	Acetolactate synthase I/II/III large subunit [EC:2.2.1.6] Acetolactate synthase I/III small subunit [EC:2.2.1.6] Acetolactate synthase II small subunit [EC:2.2.1.6] Acetolactate decarboxylase [EC:4.1.1.5] (R,R)-butanediol dehydrogenase/meso-butanediol Dehydrogenase/diacetyl reductase [EC:1.1.1.4 1.1.1.- 1.1.1.303] meso-butanediol dehydrogenase/(S,S)-butanediol dehydrogenase/diacetyl reductase [EC:1.1.1.- 1.1.1.76 1.1.1.304]	Nielsen et al., 2010
2,3-Butanediol	*E2.2.1.6L E2.2.1.6S ilvM alsD BDH butA, budC*	Acetolactate synthase I/II/III large subunit [EC:2.2.1.6] Acetolactate synthase I/III small subunit [EC:2.2.1.6] Acetolactate synthase II small subunit [EC:2.2.1.6] Acetolactate decarboxylase [EC:4.1.1.5] (R,R)-butanediol dehydrogenase/meso-butanediol dehydrogenase/diacetyl reductase [EC:1.1.1.4 1.1.1.- 1.1.1.303] Meso-butanediol dehydrogenase/(S,S)-butanediol dehydrogenase/diacetyl reductase [EC:1.1.1.- 1.1.1.76 1.1.1.304]	Nielsen et al., 2010

### Visualization of Figures

**FIGURE 1:**
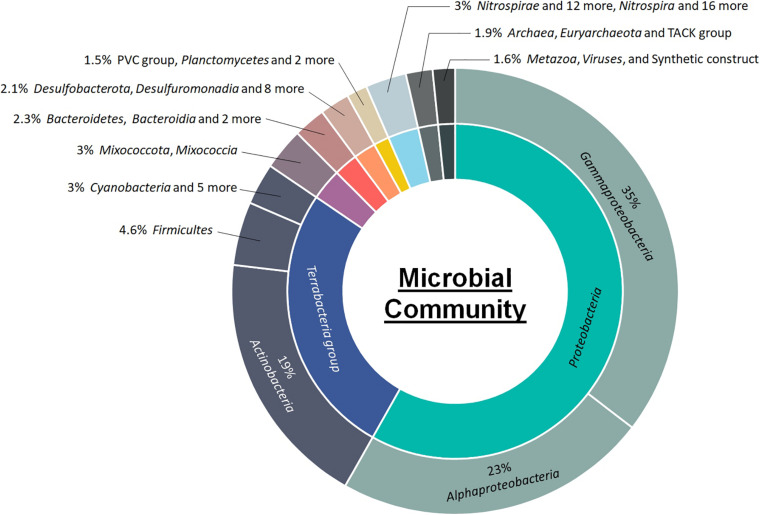
Class-level microbial community composition and relative abundance in activated sludge of landfill leachate.

**FIGURE 2:**
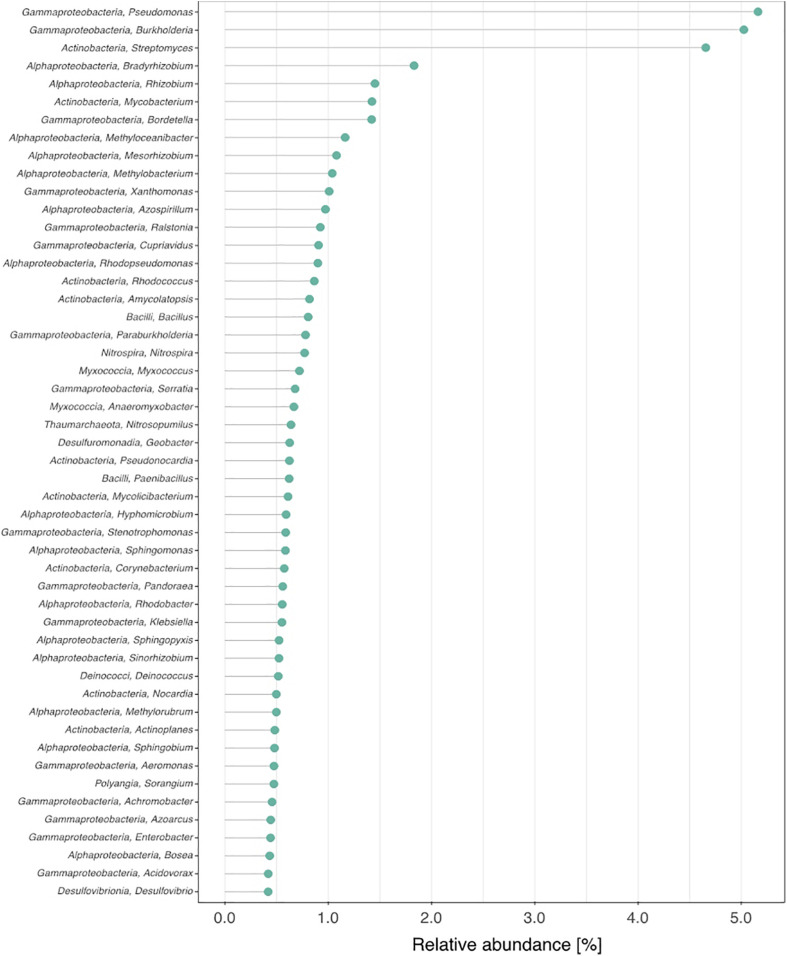
Fifty most abundant microbial genera and their relative abundances in the activated sludge sample.

**FIGURE 3:**
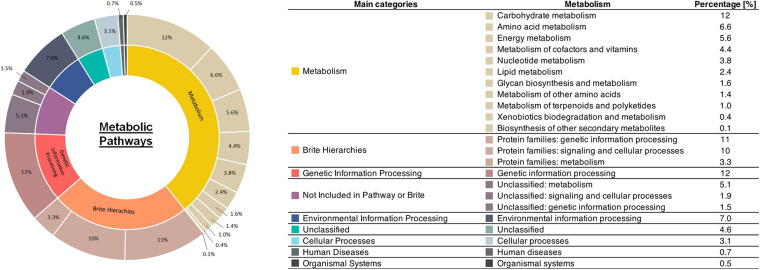
Repertories of metabolic pathways possessed by the activated sludge sample.

Taxonomic classification results (Figures 1, 2 and Supplementary Figure 1) and repertories of metabolic pathways (Figure 3) were visualized with R (R Core Team, 2020), Rstudio (RStudio_Team, 2020), and Tidyverse package (Wickham et al., 2019).

### Nucleotide Accession Numbers

The sequencing data generated by metagenomic analysis have been deposited in DDBJ with the following accession numbers: DRA009549, BioProject: PRJDB9252, and BioSample: SAMD00202538.

## Results

### Landfill Leachate Compositions and Reactor Performances

The SBR-based activated sludge system in this study intermittently received methanol as an external electron donor to replenish the limited amount of organic carbon constituents (average COD concentration of 43.9 ± 9.2 mg/L) from the closed landfill. The leachate contained a chloride concentration of 7,693 ± 1,767 mg/L, and the salinity was calculated at 1.23% by the correlation with electron conductivity and temperature (APHA et al., 1992), which is classified as brackish (Table 2). The average removal efficiencies of COD (including the added methanol) and nitrogen were 95.5 ± 1.6 and 97.2 ± 2.1%, and the volumetric removal rates of COD and T-N were 26.1 ± 8.8 g-COD/m^3^/day and 16.6 ± 3.1 g-N/m^3^/day, respectively. Nitrification was nearly complete in the SBR, resulting in an effluent ammonium concentration of below 0.05 mg N/L on average.

### Microbial Community of Mixed Culture Biomass in Activated Sludge

The class-level taxonomic composition of the activated sludge biomass is summarized in Figure 1. The class *Gamma-proteobacteria* was most abundant, accounting for up to 35% of the total population. This was followed by *Alpha-proteobacteria* (23%), and *Actinobacteria* (19%). The top 50 most abundant microbes at the genus and species levels and the corresponding relative abundances are shown in Figure 2 and Supplementary Figure 1, respectively. *Methyloceanibacter caenitepidi*, which is a marine methanol-assimilating bacterium, was the most abundant in the biomass (Supplementary Figure 1). Based on read numbers, its relative abundance was 1.3 times (1.2%) higher than that of the second most predominant species, *Rhodopseudomonas palustris* (0.9%). As for major guilds for ammonia oxidation, the genera *Candidatus* Nitrosopumilus and *Nitrospira*, known as ammonia-oxidizing archaea and complete ammonia-oxidizing bacteria, respectively, were detected (Figure 2 and Supplementary Figure 1).

### Metabolic Pathways and Genetic Production Potential of the Mixed Culture

**FIGURE 4:**
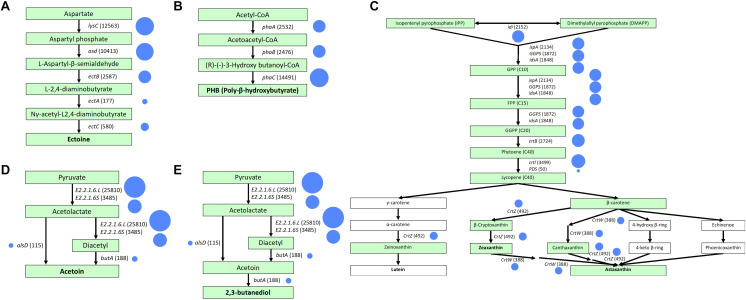
Gene-mapping to KEGG reference pathways for valuable products including **(A)** ectoine, **(B)** PHB, **(C)** lutein, zeaxanthin, and astaxanthin, **(D)** acetoin, and **(E)** 2,3-butanediol. A continuous arrow shows biosynthesis pathways. Chemical substances in boxes are generated by microbial reactions. Boxes in green indicate the presence of the metabolic reaction. Functional genes and the hit numbers are indicated next to the arrows. Circle size corresponds to the number of hits.

The results of mapping our sample to reference metabolic pathways in the KEGG are summarized in Figure 3. The highest relative gene abundance was associated with carbohydrate metabolism (12%) and genetic information processing (12%), followed by protein families: genetic information processing (11%), and protein families: signaling and cellular processes (10%). The biosynthesis pathways for each valuable product after mapping with mixed culture data are illustrated in Figure 4. The mixed culture biomass in the activated sludge possessed a full set of functional genes to synthesize six of the valuable products, ectoine, PHB, zeaxanthin, astaxanthin, acetoin, and 2,3-butanediol (Figure 4 and Table 4). Comparing the hit numbers of the functional genes for ectoine, acetoin, and 2,3-butanediol for each enzymatic reaction step (Figures 4A,D,E) revealed a substantial difference in hit numbers. Specifically, the lowest and highest gene hit numbers for ectoine biosynthesis were *ectA* (177) and *lysC* (12,563) for conversion of *L*-2,4-diaminobutyrate into *N*-γ-acetyl-L2,4,-diaminobutyrate and of aspartate into aspartyl phosphate, respectively. These hit numbers were used to designate the degree of the rate-limiting reaction for biosynthesis of a valuable product (e.g., 0.014 = 177/12,563). Acetoin and 2,3-butanediol had the common functional gene *alsD*, which had a considerably lower hit number (115) than the others, leading to a relative abundance of the lowest/highest gene hit number being 0.0045. In contrast, PHB, zeaxanthin, and astaxanthin had relatively evenly-distributed gene hit numbers for their enzymatic reactions. For PHB biosynthesis, the hit numbers of *phaA* (2,532) and *phaB* (2,476), which encode acetyl-CoA acetyltransferase and acetoacetyl-CoA reductase, respectively, were lower than the one of *phaC* (14,491), which encodes poly (3-hydroxyalkanoate) polymerase. These hit numbers resulted in relative abundances of the lowest/highest gene hit number of 0.175 (*phaA*/*phaC*) and 0.171 (*phaB*/*phaC*), respectively. The same trend appeared with zeaxanthin and astaxanthin syntheses, resulting in a relative abundance of the lowest/highest hit number of 0.11. Terpenoid backbone biosynthesis pathways after gene-mapping are shown in Supplementary Figure 2. In addition, the results of gene-mapping of each valuable product to the KEGG reference pathways are shown in Supplementary Figures 3–5 and those of the terpenoid backbone biosynthesis are shown in Supplementary Figure 6. The mapping results shown in the figures indicate that the pathways for the production of the compounds are connected.

**TABLE 4:** Potential for production of valuable products by mixed microbial communities and a single species of bacteria.

Valuable products	Mixed culture	Single bacterium	
		Lineage	Species
Ectoine	+	−	−
PHB	+	*Alphaproteobacteria; Rhizobiales Alphaproteobacteria; Rhizobiales; Hyphomicrobiaceae Gammaproteobacteria; Burkholderiales; Oxalobacteraceae*	*Methyloceanibacter caenitepidi Candidatus* Filomicrobium marinum W *Janthinobacterium* sp. Marseille
Lutein	−	−	−
Zeaxanthin	+	−	−
Astaxanthin	+	−	−
Acetoin	+	*Chloroflexi; Ardenticatenia; Ardenticatenales; Ardenticatenaceae Chloroflexi; Caldilineae; Caldilineales; Caldilineaceae Gammaproteobacteria; Enterobacterales; Pectobacteriaceae Gammaproteobacteria; Burkholderiales; Alcaligenaceae*	*Candidatus* Promineofilum breve *Caldilinea aerophila Lonsdalea britannica Orrella dioscoreae*
2,3-Butanediol	+	*Chloroflexi; Caldilineae; Caldilineales; Caldilineaceae Gammaproteobacteria; Enterobacterales; Pectobacteriaceae Gammaproteobacteria; Burkholderiales; Alcaligenaceae*	*Caldilineaaerophila Lonsdalea britannica Orrelladioscoreae*

### Genetic Potential of a Single Bacterium to Produce Valuable Compounds

Linking the phylogeny with functions revealed that PHB, acetoin, and 2,3-butanediol can be genetically produced by a single bacterial species (Table 4). However, individual species of bacteria lack certain functional genes for the production of ectoine, lutein, zeaxanthin, and astaxanthin, implying that syntrophic interactions between different microbial species are needed for synthesis of these compounds. *M. caenitepidi*, *Janthinobacterium* sp. Marseille, and *Ca. Filomicrobium marinum* W harbored *phaABC* genes encoding enzymes for PHB biosynthesis, while *Ca.* promineofilum breve, *Caldilinea aerophila*, *Lonsdalea britannica*, and *Orrella dioscoreae*, possessed genes essential to acetoin production, i.e., *ilvM, alsD*, and *butA* (Figure 4). Among the bacteria capable of producing acetoin, *Caldilinea aerophila*, *Lonsdalea britannica*, and *Orrella dioscoreae* harbor *butA* and *BDH*, which genetically produce 2,3-butanediol from acetoin.

## Discussion

### Gene-Centric Approach That Facilitates the Development of Upcycling Bioprocesses From Landfill Leachates

The MRM concept (Verstraete, 2007) paves the way for a paradigm shift in which wastewater can be regarded as a resource for valuable products. Applying a bottom-up approach based on gene-centric analysis to the MRM concept is essential for the feasibility of valuable product biosynthesis based on metabolic network information. Therefore, feasibility assessments based on the metagenome will help to establish innovative bioprocesses for valuable product biosynthesis. However, this approach has not been applied to a bioreactor for nitrification and denitrification in landfill leachate containing high concentrations of ammonia (e.g., several hundred mg N/L) and salt (above > 1%; Díaz et al., 2019; Shu et al., 2019). This study, therefore, advocates a rapid screening procedure based on the gene-centric analysis from metagenomic data in a simple manner. To the best of our knowledge, this is the first metagenomics study that investigates the metabolic potential for valuable product biosynthesis by activated sludge used for leachate treatment at a closed landfill site. The application of gene-centric metagenomic analysis confirmed that the activated sludge possessed the metabolic potential for the biosynthesis of six targeted products. The holistic and exclusive screening of metabolic pathway networks for valuable products based on gene-centric metagenomic analysis revealed (i) the potential for valuable product biosynthesis (Table 4) and (ii) inferred potential rate-limiting steps within the metabolic pathways (Figure 4). Although the taxonomic and functional information attained by the gene-centric analysis is not as profound as that by the genome-centric counterpart, the information provides the implication if a biomass sample possesses metabolic potentials for valuable product production or not. The gene-centric approach implemented in this study will facilitate the development of upcycling bioprocesses from landfill leachates in which organic carbon and nitrogen are abundant.

The gene hit number analysis suggests a potential rate-limiting step during the bio-transformations for value-added compounds, e.g., the conversion from *L*-2,4-diaminobutylate to *N*-γ-acetyl-L2,4-diaminobutyrate in a series of ectoine production from aspartate (Figure 4). A challenge in this study stems from the authenticity of the gene hit number as an indicator for rate-limiting steps in the metabolic pathways because the differences in gene expression affecting RNA and protein abundances, and the existence of pseudogenes may also determine enzymatic reactions. Although this challenge is under debate, comparing the gene hit numbers may provide rough measures of bottleneck enzymes in metabolic reactions. The credibility of quantifying gene hit numbers warrants future investigation.

### Metabolic Potentials of Activated Sludge Biomass at a Landfill Leachate Treatment Plant

This study demonstrated that the mixed microbial community in a landfill leachate treatment site harbored functional genes associated with the syntheses of ectoine, PHB, zeaxanthin, astaxanthin, acetoin, and 2,3-butanediol (Table 4). The presence of these genes suggests the potential for use of this activated sludge biomass in biosynthesis. As previously demonstrated, microbial consortia in activated sludge have been shown to produce short-chain fatty acids (Li et al., 2014), enzymes (Yu et al., 2009), polyhydroxyalkanoate (PHA; Lee and Yu, 1997), bio-flocculants (Wang et al., 2007), and fuels (Salama et al., 2017). As for ectoine synthesis, the analysis of the hit numbers of each functional gene suggested the conversion of *L*-2,4-diaminobutylate into *N*-γ-acetyl-L2,4-diaminobutyrate mediated by *L*-2,4-diaminobutyric acid acetyltransferase (EctA) as a rate-limiting step. Previous studies of EctA purified from halophilic bacteria revealed that suitable conditions for this process were pH 8.2–9.5, a temperature of 20°C, and a salt concentration of 2.3% (Ono et al., 1999; Richter et al., 2020), which do not coincide with the SBR conditions in this study (pH 7.35 ± 0.08, temperature of 28.8°C ± 1.4°C, and salinity of 1.23%). Moreover, caution should be taken when considering these findings because the conditions required to activate EctA *in vitro* and *in vivo* may not be the same. Accordingly, future studies to identify suitable environmental conditions for bacteria possessing EctA are needed.

### Potential Valuable Products Produced by a Single Microorganism

The metagenomic analysis of the activated sludge used to treat landfill leachate allowed classification of the production potential for valuable products by (i) individual species of bacteria or (ii) mixed microbial communities (Table 4). The results demonstrated that individual species of bacteria present in the activated sludge of the landfill leachate treatment had the genetic potential to synthesize PHB, acetoin, and 2,3-butanediol among the seven products listed in Table 4.

Our metagenomic analysis unraveled that the species proximal to *M. caenitepidi*, *Ca. Filomicrobium marinum* W, and *Janthinobacterium* sp. Marseille were all found to possess the entire set of genes encoding for PHB synthesis. The metagenomic analysis of the taxonomy and functions revealed for the first time that *M. caenitepidi*, a marine methanol-assimilating bacterium, was dominant in the activated sludge of the landfill leachate treatment facility (Supplementary Figure 1). The presence of *M. caenitepidi* aside from the ocean has not been reported, except for one study (Yasuda et al., 2020). *M. caenitepidi* Gela4 was isolated from marine sediment near a hydrothermal vent containing methane in Kagoshima Bay, Japan (Takeuchi et al., 2014) and from a Belgian North Sea sediment (Vekeman et al., 2016). The genus Filomicrobium was detected in a methanol-fed denitrifying bioreactor from saline water (Rissanen et al., 2016). *Ca. Filomicrobium marinum* W (Henriques and De Marco, 2015), which was first isolated from North Atlantic surface seawater, is a methylotroph capable of potentially synthesizing PHB. *Janthinobacterium* isolates acquired from the Antarctic possess functional genes for PHB biosynthesis. Although they thrive in marine environments (Tan et al., 2020), the detection of *Janthinobacterium* sp. as a PHB producer in the landfill leachate treatment site makes sense as the presence of methanol and salinity provide a comparable environment. These species require greater ecophysiological descriptions to leverage their PHB synthesis potentials, which will be a follow-up aim.

Bacteria harboring *phaABC* store PHB intracellularly under feast-famine conditions (Inoue et al., 2018). Because the SBR in this study may induce feast–famine conditions over one cycle, these three bacteria accumulated PHB to attain energy for their preferential growth. Accordingly, the conditions likely exerted high selection pressure to preferentially enrich the sludge with these three potential PHB producers. Identification of their presence and functions paves the way for developing a PHB-producing process in a water/wastewater treatment system. However, this preliminary study was designed to identify the functions and compositions of an activated sludge microbiome treating landfill leachate, not to quantify water and intracellular constituents in the SBR. Therefore, future investigations to evaluate long-term SBR operation and the associated rates of production of valuable products in accordance with microbial population dynamics in the activated sludge are warranted.

This study also revealed that the bacteria listed in Table 4 harbor genes capable of producing acetoin and 2,3-butanediol. However, the ecological niches of taxonomic proxies for these bacteria are not consistent. *Ca.* Promineofilum and *Caldilinea aerophile*, which belong to the phylum *Chloroflexi*, are commonly found in municipal wastewater treatment plants (McIlroy et al., 2016) and hot spring sulfur turf mats (Sekiguchi et al., 2003), while *Lonsdalea Britannica* and *Orrella dioscoreae* are *Gamma-proteobacteria* isolated from the leaves of *Quercus robur* (Li et al., 2017) and *Dioscorea sansibarensis* (Carlier et al., 2017), respectively. Despite their genetic potentials to produce valuable products, in-depth physiological activity tests for actual production have not been conducted (Sekiguchi et al., 2003; Brady et al., 2012; McIlroy et al., 2016; Carlier et al., 2017; Li et al., 2017). Future research will be undertaken to confirm the product availability, yield, and production rate by bacterial species harboring genes related to PHB, acetoin, and 2,3-butanediol production.

### Metagenomic Screening of Promising Bacteria Capable of Producing Valuable Products

**FIGURE 5:**
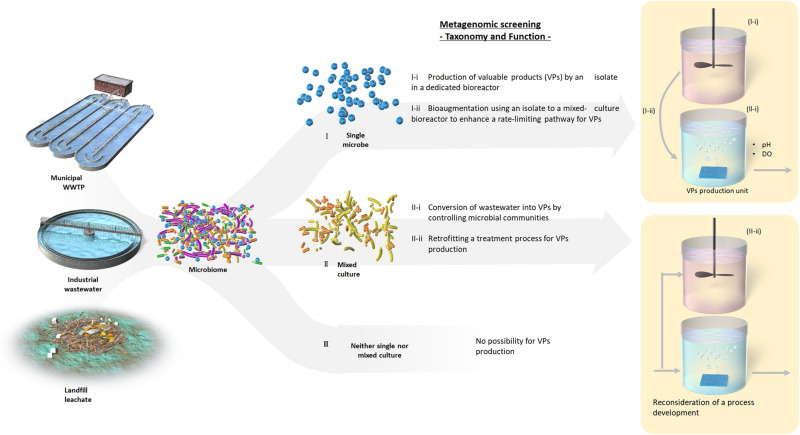
A workflow of a gene-based metagenomic screening method for production of valuable products.

In-depth analysis of mixed microbial community compositions and functions by gene-centric metagenomic analysis, proposed in this study, could facilitate a primary screening strategy for exploration and leveraging of promising microbial communities or species. Our gene-centric analysis confirmed that the activated sludge investigated did not harbor the functional genes encoding enzymes to synthesize lutein (Supplementary Figure 5). Moreover, inoculating the activated sludge would not succeed in enrichment with a microbial guild producing lutein. Pre-screening by gene-centric metagenomic analysis allows determination of whether a single microbe (I), a mixed microbial community (II), or no microbes (III) synthesize a valuable product (Figure 5). Such classifications may enable identification of the conditions required for a key mixed microbial community or an individual microbe to be enriched (Cases I and II Figure 5), ultimately allowing the development of an innovative microbial process. Accordingly, implementing bioaugmentation strategies (Laocharoen et al., 2015; Lin et al., 2018) or retrofitting treatment processes for valuable product biosynthesis, analogous to the concept of bioaugmentation technology where nitrifying bacteria are enriched in a tank installed in a sludge recycle line (Salem et al., 2003), are potential applications of this screening process (Figure 5). These findings will also enable research toward isolation or selective enrichment of promising microbes using suitable environmental conditions identified from metagenomic data. Moreover, if screening reveals no microbes have the desired properties, the method developed here will save a great deal of time and labor. The advocated scheme could be beneficial especially for engineers to bridge fundamental and applied research toward resource recovery and upcycling.

## Conclusion

In this study, gene-centric metagenomic analysis of an activated sludge used to treat landfill leachate with a high nitrogen concentration and methanol supplementation revealed the genetic potential for biosynthesis of valuable products by microorganisms in the activated sludge. Specifically, it was confirmed that PHB, ectoine, astaxanthin, zeaxanthin, acetoin, and 2,3-butanediol could be biosynthesized by the mixed microbial community in the activated sludge. Moreover, the pathway-based analysis implied a rate-limiting step for each product. PHB, acetoin and 2,3-butanediol also had the biosynthetic potential to be produced by individual species of microorganisms in the activated sludge. Furthermore, a marine methanol-assimilating bacterium with the genetic potential for PHB biosynthesis, *M. caenitepidi*, was shown to be dominant in the activated sludge. Our results demonstrate the potential for application of this method as tool for primary screening of promising microbial species in development of bioprocesses for valuable product biosynthesis.

Overall, primary screening by gene-centric metagenomic analysis can be used to evaluate the potential for valuable compound production by microbial communities or individual species in engineered systems was demonstrated. The analysis specifically provides engineers with an opportunity to preliminarily test a possibility of mixed culture biomass to produce valuable products. Future studies to link the screening by metagenomic analysis as described in this study with enrichment and isolation of promising microbial species are warranted.

## Data Availability Statement

The datasets presented in this study can be found in online repositories. The names of the repository/repositories and accession number(s) can be found in the article/Supplementary Material.

## Author Contributions

AT designed and led the study. SY and AT took samples and extracted DNA. SY, LO, and SA conducted the genomic and metabolic pathway analyses. SY, TS, LO, and SA performed laboratory works for metagenomic analysis. SY wrote the manuscript with major edits. SL and AT discussed the outline of the manuscript and edited the manuscript. All authors contributed to the article and approved the submitted version.

## Conflict of Interest

The authors declare that the research was conducted in the absence of any commercial or financial relationships that could be construed as a potential conflict of interest.
