# Supplementary material for: Metagenomic Insights Into Functional and Taxonomic Compositions of an Activated Sludge Microbial Community Treating Leachate of a Completed Landfill: A Pathway-Based Analysis
Source: Front Microbiol. 2021 Apr 30;12:640848. doi: 10.3389/fmicb.2021.640848 (PMC8121002; doi:10.3389/fmicb.2021.640848)
Supplement: Supplementary file 1 [file Data_Sheet_1.PDF]

SUPPLEMENTARY INFORMATION

Title

Metagenomic insights into functional and taxonomic compositions of an activated sludge microbial community treating leachate of a completed landfill: a pathway-based analysis

Authors

Shohei Yasuda[1], Toshikazu Suenaga[2], Laura Orschler[3], Shelesh Agrawal[3], Susanne Lackner[3], Akihiko Terada[1,2*]

1. Department of Chemical Engineering, Tokyo University of Agriculture and Technology, 2-24-16 Naka-Cho, Koganei, Tokyo, 184-8588, Japan

2. Global Innovation Research Institute, Tokyo University of Agriculture and Technology, 3-8-1 Harumi-Cho, Fuchu, Tokyo, 185-8538, Japan

3. Department of Civil and Environmental Engineering Science, Institute IWAR, Chair of Wastewater Engineering, Technical University of Darmstadt, Franziska-Braun-Straße 7, 64287 Darmstadt, Germany

* Corresponding: A. Terada (akte@cc.tuat.ac.jp)

FIGURE LEGENGS

Table S1 Classification and application of valuable products.

Table S2 Carotenoids and terpenoids biosynthesis related functional genes.

Fig. S1 Fifty most abundant microbial species in the activated sludge sample and read numbers.

Fig. S2 Terpenoid backbone biosynthesis pathways. An arrow of continuous line shows biosynthesis pathways. Chemical substances in boxes are generated materials by metabolism. Boxes colored in green indicates that the metabolism reaction is taken place. Functional genes and the hit number are shown sideways of the arrow. Circle size indicates sizes of the hit number.

Fig. S3 Gene-mapping to KEGG reference pathways for Ectoine.

Fig. S4 Gene-mapping to KEGG reference pathways for PHB, Acetoin, and 2,3-butanediol.

Fig. S5 Gene-mapping to KEGG reference pathways for Zeaxanthin, Astaxanthin, and Lutein.

Fig. S6 Gene-mapping to KEGG reference pathways of Terpenoid backbone biosynthesis

Table S1

Valuable products	Classification	Application	Citation
PHB (polyhydroxybutylate)	Biocompatible and biodegradable resin	Bottles<br>Suture threads	Anderson et al, 1990<br>Levett et al, 2016
ectoine	Osmolytes	Cosmetics	Berry et al, 2003<br>Cantera et a, 2016
lutein	Carotenoids	Supplements for eyes	Zhang, 2018
zeaxanthin	Carotenoids	Supplements for eyes	Zhang, 2018
astaxanthin	Carotenoids	Supplements for eyes	Zhang, 2018
acetoin	Hydroxyketone	Food additives	Xiao and Lu, 2014
2,3-butanediol	Diol	Platform chemical<br>(Raw materials of MEK and 1,3-butadiene)	Celińska et al, 2009

Table S2

Catergory	KeggID	Gene Name	Name	EC No.
Carotenoid	K02291	*crtB*	15-cis-phytoene synthase	EC:2.5.1.32
	K02293	*PDS*	15-cis-phytoene desaturase	EC:1.3.5.5
	K09836	*crtW*	beta-carotene/zeaxanthin 4-ketolase	EC:1.14.99.641.14.99.63
	K15746	*crtZ*	beta-carotene 3-hydroxylase	EC:1.14.15.24
	K01823	*idi*	isopentenyl-diphosphate Delta-isomerase	EC:5.3.3.2
	K00795	*ispA*	farnesyl diphosphate synthase	EC:2.5.1.10 2.5.1.1
	K13789	*GGPS*	geranylgeranyl diphosphate synthase, type II	EC:2.5.1.292.5.1.10 2.5.1.1
	K13787	*idsA*	geranylgeranyl diphosphate synthase, type I	EC:2.5.1.292.5.1.10 2.5.1.1
Terpenoids	K01662	dxs	1-deoxy-D-xylulose-5-phosphate synthase	EC:2.2.1.7
	K00099	ispC	1-deoxy-D-xylulose-5-phosphate reductoisomerase	EC:1.1.1.267
	K00991	ispD	2-C-methyl-D-erythritol 4-phosphate cytidylyltransferase	EC:2.7.7.60
	K12506	ispDF	2-C-methyl-D-erythritol 4-phosphate cytidylyltransferase / 2-C-methyl-D-erythritol 2,4-cyclodiphosphate synthase	EC:4.6.1.12 2.7.7.60
	K00919	ispE	4-diphosphocytidyl-2-C-methyl-D-erythritol kinase	EC:2.7.1.148
	K01770	ispF	2-C-methyl-D-erythritol 2,4-cyclodiphosphate synthase	EC:4.6.1.12
	K03526	gcpE	(E)-4-hydroxy-3-methylbut-2-enyl-diphosphate synthase	EC:1.17.7.3 1.17.7.1
	K03527	ispH	4-hydroxy-3-methylbut-2-en-1-yl diphosphate reductase	EC:1.17.7.4
	K00626	E2.3.1.9	acetyl-CoA C-acetyltransferase	EC:2.3.1.9
	K01641	E2.3.3.10	hydroxymethylglutaryl-CoA synthase	EC:2.3.3.10
	K00021	HMGCR	hydroxymethylglutaryl-CoA reductase (NADPH)	EC:1.1.1.34
	K00054	mvaA	hydroxymethylglutaryl-CoA reductase	EC:1.1.1.88
	K00869	E2.7.1.36	mevalonate kinase	EC:2.7.1.36
	K00938	E2.7.4.2	phosphomevalonate kinase	EC:2.7.4.2
	K01597	MVD	diphosphomevalonate decarboxylase	EC:4.1.1.33
	K01823	idi	isopentenyl-diphosphate Delta-isomerase	EC:5.3.3.2

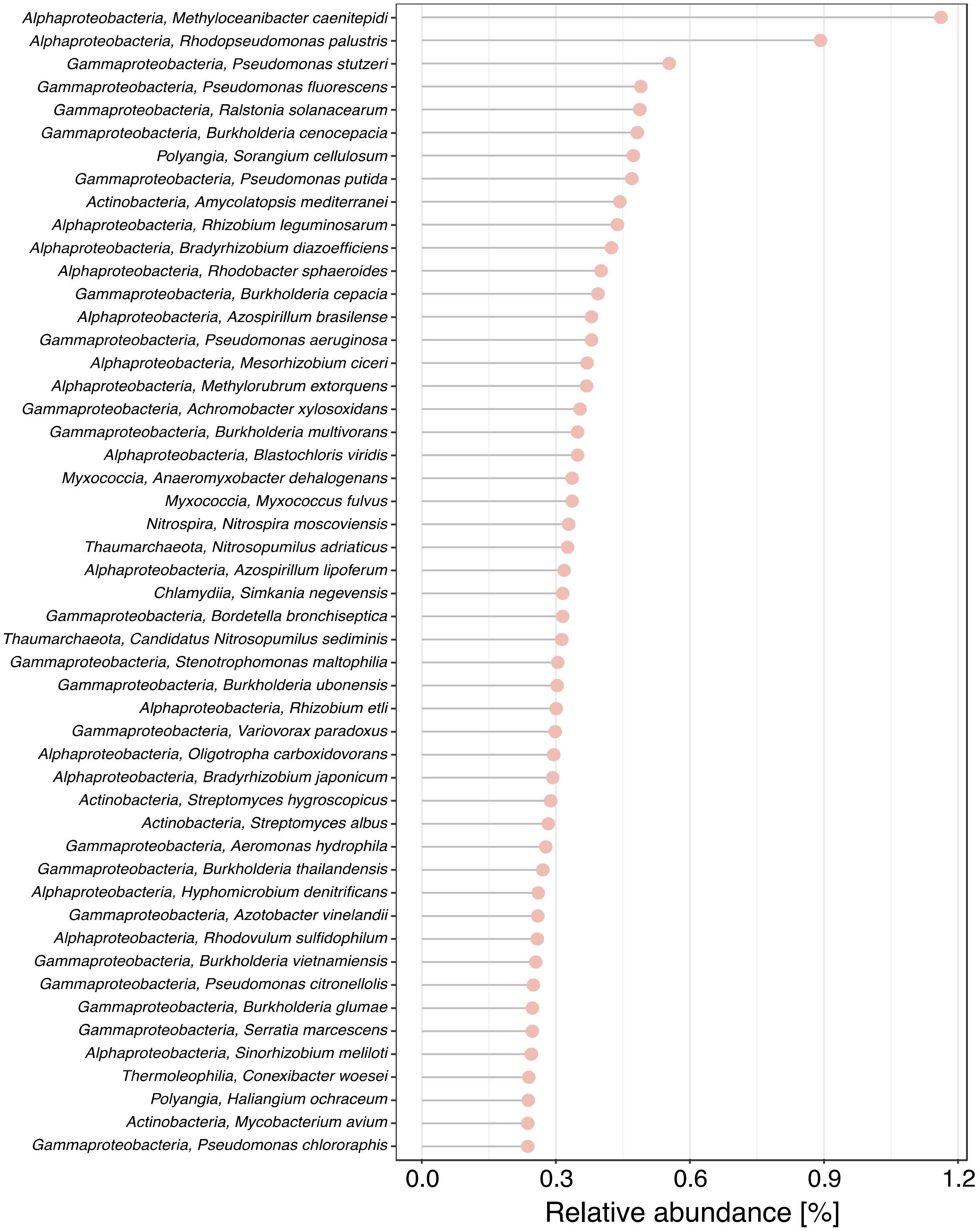
Alphaproteobacteria, Methyloceanibacter caenitepidi
Alphaproteobacteria, Rhodopseudomonas palustris
Gammaproteobacteria, Pseudomonas stutzeri
Gammaproteobacteria, Pseudomonas fluorescens
Gammaproteobacteria, Ralstonia solanacearum
Gammaproteobacteria, Burkholderia cenocepacia
Polyangia, Sorangium cellulosum
Gammaproteobacteria, Pseudomonas putida
Actinobacteria, Amycolatopsis mediterranei
Alphaproteobacteria, Rhizobium leguminosarum
Alphaproteobacteria, Bradyrhizobium diazoefficiens
Alphaproteobacteria, Rhodobacter sphaeroides
Gammaproteobacteria, Burkholderia cepacia
Alphaproteobacteria, Azospirillum brasilense
Gammaproteobacteria, Pseudomonas aeruginosa
Alphaproteobacteria, Mesorhizobium ciceri
Alphaproteobacteria, Methylorubrum extorquens
Gammaproteobacteria, Achromobacter xylosoxidans
Gammaproteobacteria, Burkholderia multivorans
Alphaproteobacteria, Blastochloris viridis
Myxococcia, Anaeromyxobacter dehalogenans
Myxococcia, Myxococcus fulvus
Nitrospira, Nitrospira moscoviensis
Thaumarchaeota, Nitrosopumilus adriaticus
Alphaproteobacteria, Azospirillum lipoferum
Chlamydiia, Simkania negevensis
Gammaproteobacteria, Bordetella bronchiseptica
Thaumarchaeota, Candidatus Nitrosopumilus sediminis
Gammaproteobacteria, Stenotrophomonas maltophilia
Gammaproteobacteria, Burkholderia ubonensis
Alphaproteobacteria, Rhizobium etli
Gammaproteobacteria, Variovorax paradoxus
Alphaproteobacteria, Oligotropha carboxidovorans
Alphaproteobacteria, Bradyrhizobium japonicum
Actinobacteria, Streptomyces hygroscopicus
Actinobacteria, Streptomyces albus
Gammaproteobacteria, Aeromonas hydrophila
Gammaproteobacteria, Burkholderia thailandensis
Alphaproteobacteria, Hyphomicrobium denitrificans
Gammaproteobacteria, Azotobacter vinelandii
Alphaproteobacteria, Rhodovulum sulfidophilum
Gammaproteobacteria, Burkholderia vietnamiensis
Gammaproteobacteria, Pseudomonas citronellolis
Gammaproteobacteria, Burkholderia glumae
Gammaproteobacteria, Serratia marcescens
Alphaproteobacteria, Sinorhizobium meliloti
Thermoleophilia, Conexibacter woesei
Polyangia, Haliangium ochraceum
Actinobacteria, Mycobacterium avium
Gammaproteobacteria, Pseudomonas chlororaphis
0.0
0.3
0.6
0.9
1.2
Relative abundance [%]

Figure S1

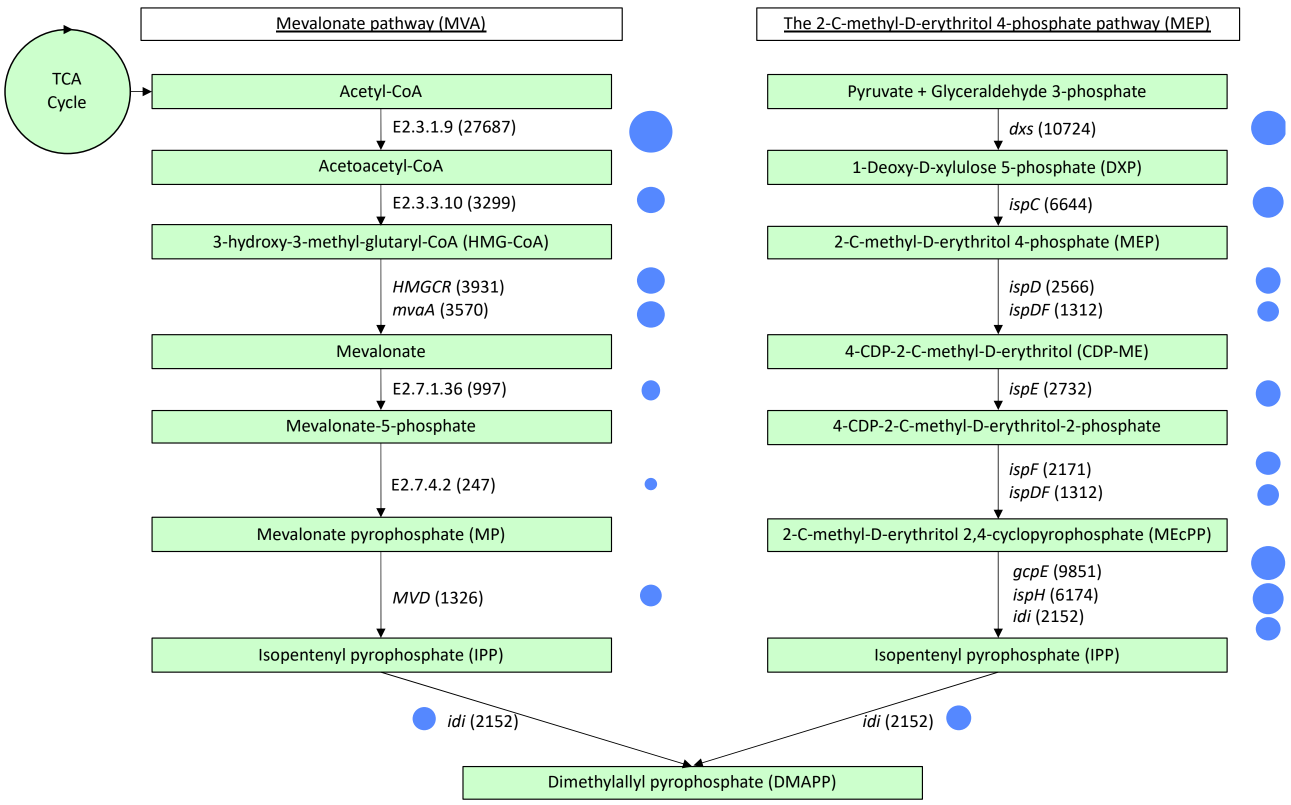

Mevalonate pathway (MVA)
The 2-C-methyl-D-erythritol 4-phosphate pathway (MEP)
TCA
Cycle
Acetyl-CoA
E2.3.1.9 (27687)
Acetoacetyl-CoA
E2.3.3.10 (3299)
3-hydroxy-3-methyl-glutaryl-CoA (HMG-CoA)
HMGCR (3931)
mvaA (3570)
Mevalonate
E2.7.1.36 (997)
Mevalonate-5-phosphate
E2.7.4.2 (247)
Mevalonate pyrophosphate (MP)
MVD (1326)
Isopentenyl pyrophosphate (IPP)
Pyruvate + Glyceraldehyde 3-phosphate
dxs (10724)
1-Deoxy-D-xylulose 5-phosphate (DXP)
ispC (6644)
2-C-methyl-D-erythritol 4-phosphate (MEP)
ispD (2566)
ispDF (1312)
4-CDP-2-C-methyl-D-erythritol (CDP-ME)
ispE (2732)
4-CDP-2-C-methyl-D-erythritol-2-phosphate
ispF (2171)
ispDF (1312)
2-C-methyl-D-erythritol 2,4-cyclopyrophosphate (MEcPP)
gcpE (9851)
ispH (6174)
idi (2152)
Isopentenyl pyrophosphate (IPP)
idi (2152)
idi (2152)
Dimethylallyl pyrophosphate (DMAPP)

Figure S2

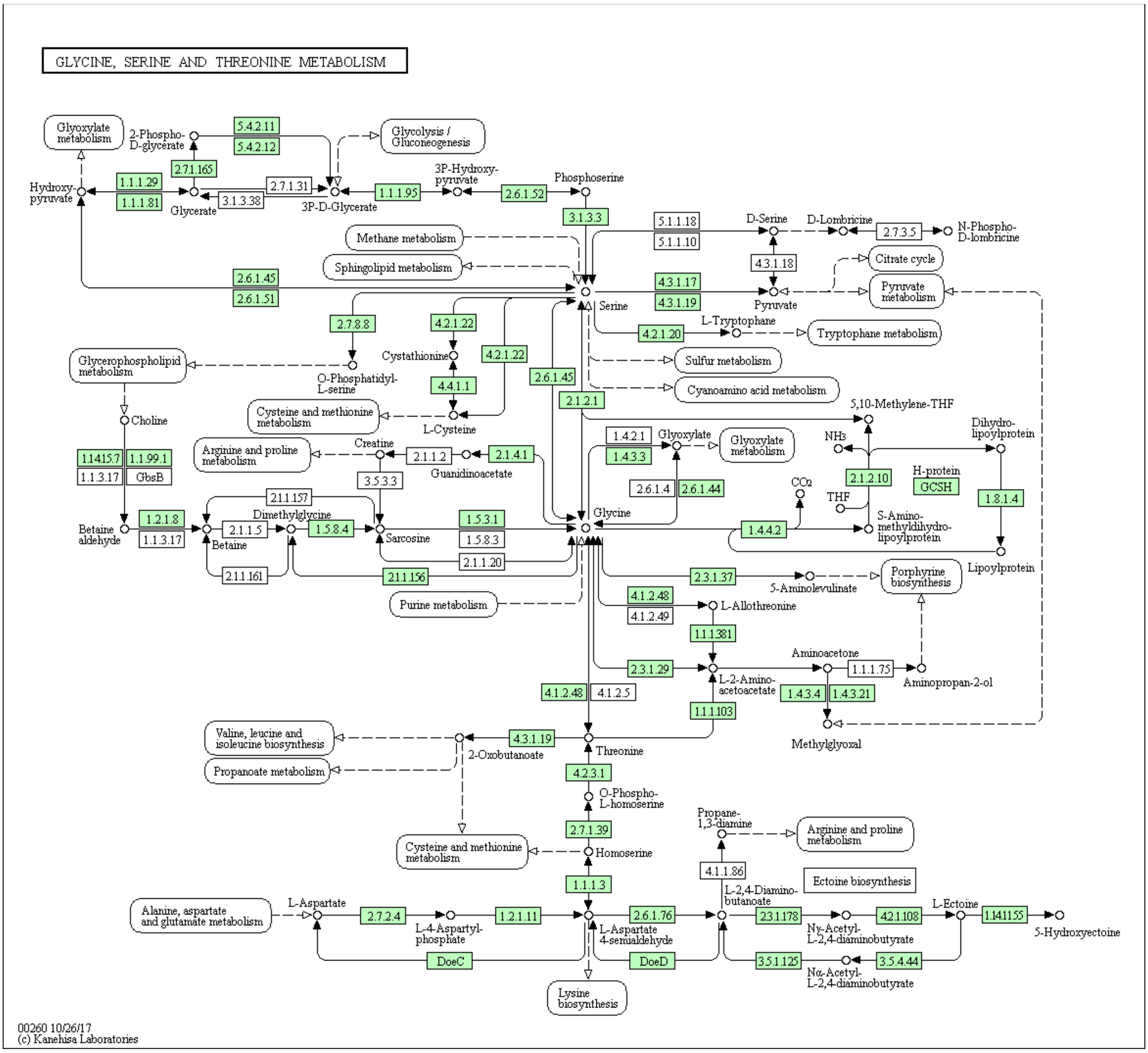
GLYCINE, SERINE AND THREONINE METABOLISM
Glyoxylate metabolism
2-Phospho-D-glycerate
5.4.2.11
5.4.2.12
Glycolysis / Gluconeogenesis
2.7.1.165
Hydroxy-pyruvate
1.1.1.29
1.1.1.81
Glycerate
2.7.1.31
3.1.3.38
3P-D-Glycerate
1.1.1.95
3P-Hydroxy-pyruvate
2.6.1.52
Phosphoserine
3.1.3.3
Methane metabolism
Sphingolipid metabolism
5.1.1.18
5.1.1.10
D-Serine
D-Lombricine
2.7.3.5
N-Phospho-D-lombricine
4.3.1.18
Citrate cycle
2.6.1.45
2.6.1.51
4.3.1.17
4.3.1.19
Serine
Pyruvate
Pyruvate metabolism
2.7.8.8
4.2.1.22
4.2.1.20
L-Tryptophane
Tryptophane metabolism
Glycerophospholipid metabolism
Cystathionine
4.2.1.22
Sulfur metabolism
O-Phosphatidyl-L-serine
2.6.1.45
4.4.1.1
Cyanoamino acid metabolism
Choline
Cysteine and methionine metabolism
L-Cysteine
2.1.2.1
5,10-Methylene-THF
1.4.2.1
Glyoxylate
Glyoxylate metabolism
Dihydro-lipoylprotein
Arginine and proline metabolism
Creatine
2.1.1.2
2.1.4.1
Guanidinoacetate
1.4.3.3
NH3
1.1.4.15.7
1.1.99.1
1.1.3.17
GbsB
3.5.3.3
2.6.1.4
2.6.1.44
CO2
2.1.2.10
H-protein
GCSH
THF
1.8.1.4
2.1.1.157
Dimethylglycine
Glycine
Betaine aldehyde
1.2.1.8
1.1.3.17
2.1.1.5
Betaine
1.5.8.4
Sarcosine
1.5.3.1
1.5.8.3
1.4.4.2
S-Amino-methyldihydro-lipoylprotein
2.1.1.20
Lipoylprotein
2.1.1.161
2.1.1.156
2.3.1.37
5-Aminolevulinate
Porphyrine biosynthesis
Purine metabolism
4.1.2.48
4.1.2.49
L-Allothreonine
1.1.1.381
2.3.1.29
Aminoacetone
1.1.1.75
L-2-Amino-acetoacetate
Aminopropan-2-ol
4.1.2.48
4.1.2.5
1.4.3.4
1.4.3.21
1.1.1.103
Methylglyoxal
Valine, leucine and isoleucine biosynthesis
4.3.1.19
Threonine
2-Oxobutanoate
Propanoate metabolism
4.2.3.1
O-Phospho-L-homoserine
Propane-1,3-diamine
Arginine and proline metabolism
2.7.1.39
Cysteine and methionine metabolism
Homoserine
4.1.1.86
Ectoine biosynthesis
1.1.1.3
L-2,4-Diamino-butanoate
Alanine, aspartate and glutamate metabolism
L-Aspartate
2.7.2.4
1.2.1.11
2.6.1.76
2.3.1.178
4.2.1.108
L-Ectoine
1.14.11.55
5-Hydroxyectoine
L-4-Aspartyl-phosphate
L-Aspartate 4-semialdehyde
Nγ-Acetyl-L-2,4-diaminobutyrate
DoeC
DoeD
3.5.1.125
3.5.4.44
Nα-Acetyl-L-2,4-diaminobutyrate
Lysine biosynthesis
00260 10/26/17
(c) Kanehisa Laboratories

Figure S3

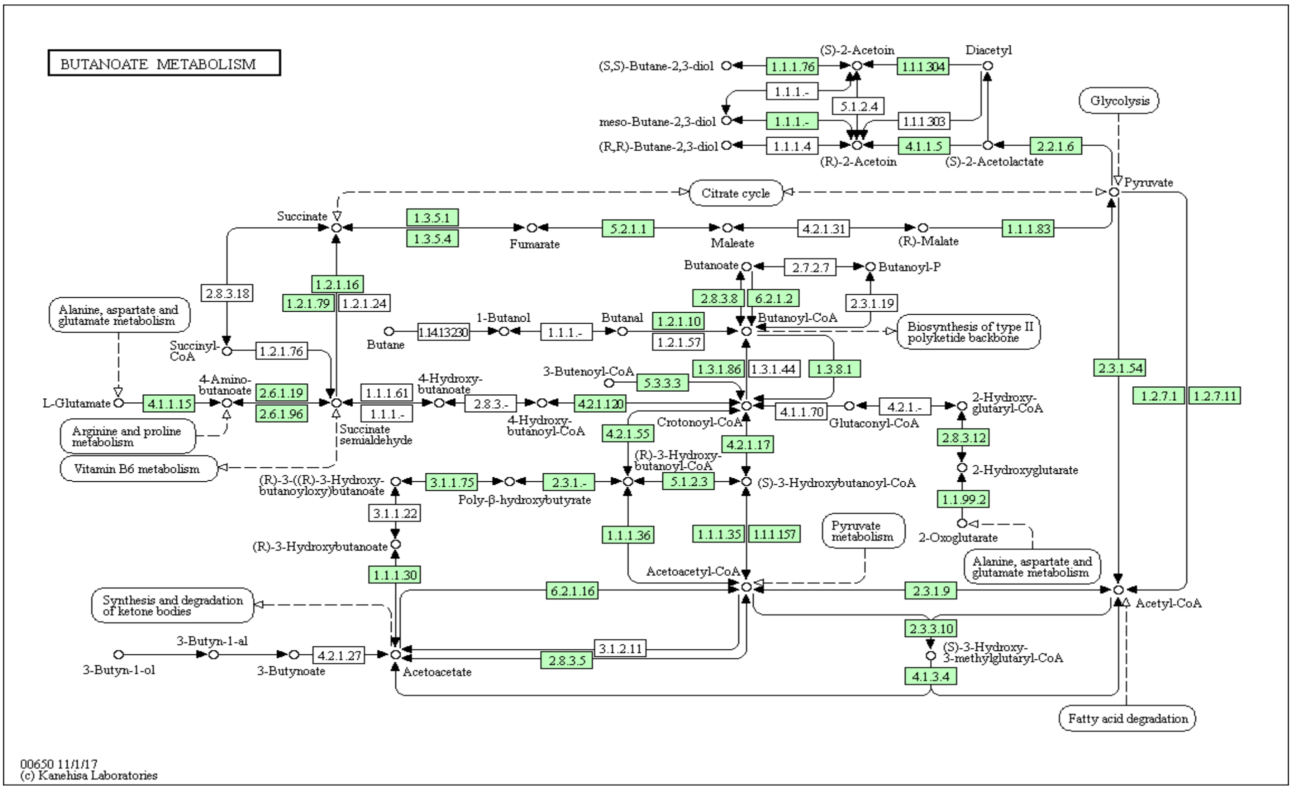
BUTANOATE METABOLISM
(S,S)-Butane-2,3-diol
(S)-2-Acetoin
Diacetyl
meso-Butane-2,3-diol
(R,R)-Butane-2,3-diol
(R)-2-Acetoin
(S)-2-Acetolactate
Glycolysis
Pyruvate
Citrate cycle
Succinate
Fumarate
Maleate
(R)-Malate
Butanoate
Butanoyl-P
Butanoyl-CoA
Alanine, aspartate and glutamate metabolism
Succinyl-CoA
1-Butanol
Butanal
Butane
Biosynthesis of type II polyketide backbone
L-Glutamate
4-Amino-butanoate
4-Hydroxy-butanoate
Succinate semialdehyde
4-Hydroxy-butanoyl-CoA
3-Butenoyl-CoA
Crotonoyl-CoA
Glutaconyl-CoA
2-Hydroxy-glutaryl-CoA
Arginine and proline metabolism
Vitamin B6 metabolism
(R)-3-((R)-3-Hydroxy-butanoyloxy)butanoate
Poly-β-hydroxybutyrate
(R)-3-Hydroxy-butanoyl-CoA
(S)-3-Hydroxybutanoyl-CoA
2-Hydroxyglutarate
(R)-3-Hydroxybutanoate
Pyruvate metabolism
2-Oxoglutarate
Acetoacetyl-CoA
Synthesis and degradation of ketone bodies
Acetyl-CoA
3-Butyn-1-al
3-Butyn-1-ol
3-Butynoate
Acetoacetate
(S)-3-Hydroxy-3-methylglutaryl-CoA
Fatty acid degradation
00650 11/1/17
(c) Kanehisa Laboratories

Figure S4

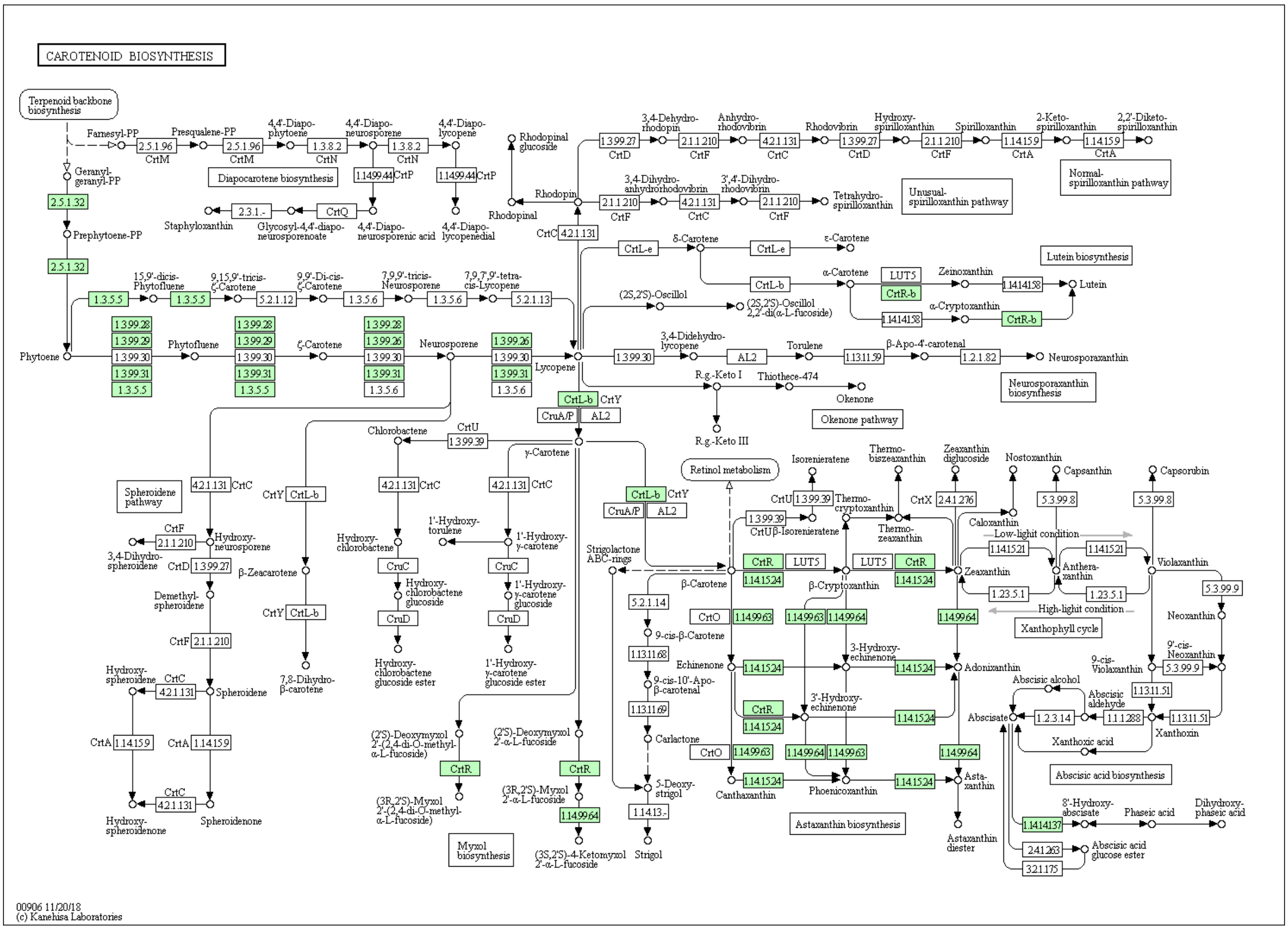
CAROTENOID BIOSYNTHESIS
Terpenoid backbone biosynthesis
Farnesyl-PP
Presqualene-PP
4,4'-Diapophytoene
4,4'-Diaponeurosporene
4,4'-Diapolycopene
Geranylgeranyl-PP
Prephytoene-PP
Phytoene
Phytofluene
ζ-Carotene
Neurosporene
Lycopene
Rhodopinal glucoside
Rhodopin
Rhodopinal
Staphyloxanthin
Diapocarotene biosynthesis
δ-Carotene
ε-Carotene
α-Carotene
Zeinoxanthin
Lutein
α-Cryptoxanthin
Lutein biosynthesis
β-Carotene
β-Cryptoxanthin
Zeaxanthin
Antheraxanthin
Violaxanthin
Neoxanthin
Xanthophyll cycle
Echinenone
Canthaxanthin
Adonixanthin
Astaxanthin
Phoenicoxanthin
Astaxanthin biosynthesis
Abscisic acid biosynthesis
Retinol metabolism
Okenone pathway
Neurosporaxanthin biosynthesis
Spheroidene pathway
Myxol biosynthesis
Strigol
00906 11/20/18
(c) Kanehisa Laboratories

Figure S5

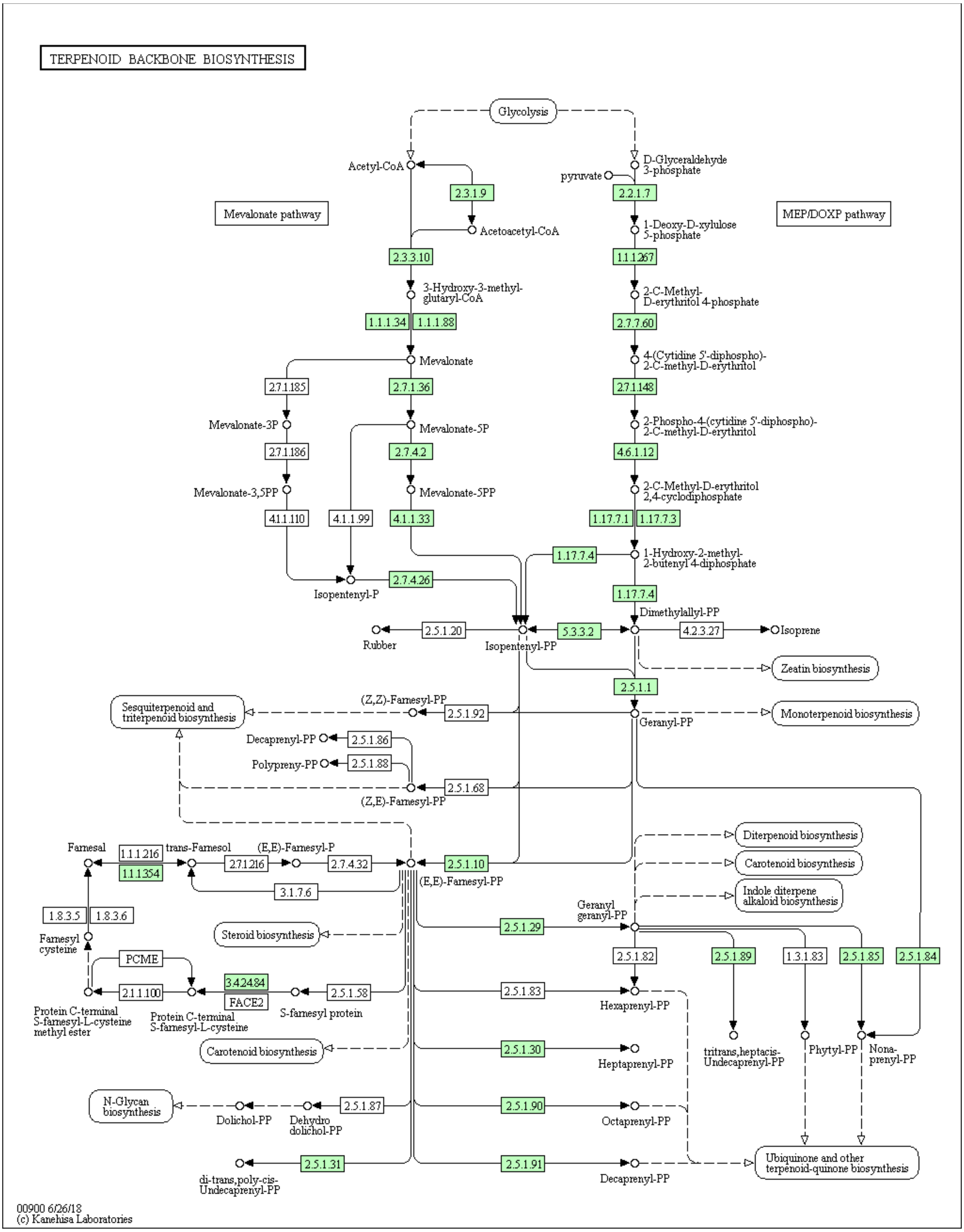

TERPENOID BACKBONE BIOSYNTHESIS
Glycolysis
Acetyl-CoA
D-Glyceraldehyde 3-phosphate
pyruvate
2.3.1.9
2.2.1.7
Mevalonate pathway
MEP/DOXP pathway
Acetoacetyl-CoA
1-Deoxy-D-xylulose 5-phosphate
2.3.3.10
1.1.1.267
3-Hydroxy-3-methyl-glutaryl-CoA
2-C-Methyl-D-erythritol 4-phosphate
1.1.1.34
1.1.1.88
2.7.7.60
Mevalonate
4-(Cytidine 5'-diphospho)-2-C-methyl-D-erythritol
2.7.1.185
2.7.1.36
2.7.1.148
Mevalonate-3P
Mevalonate-5P
2-Phospho-4-(cytidine 5'-diphospho)-2-C-methyl-D-erythritol
2.7.1.186
2.7.4.2
4.6.1.12
Mevalonate-3,5PP
Mevalonate-5PP
2-C-Methyl-D-erythritol 2,4-cyclodiphosphate
4.1.1.110
4.1.1.99
4.1.1.33
1.17.7.1
1.17.7.3
1.17.7.4
1-Hydroxy-2-methyl-2-butenyl 4-diphosphate
2.7.4.26
Isopentenyl-P
1.17.7.4
Dimethylallyl-PP
Rubber
2.5.1.20
Isopentenyl-PP
5.3.3.2
4.2.3.27
Isoprene
Zeatin biosynthesis
2.5.1.1
Sesquiterpenoid and triterpenoid biosynthesis
(Z,Z)-Farnesyl-PP
2.5.1.92
Geranyl-PP
Monoterpenoid biosynthesis
Decaprenyl-PP
2.5.1.86
Polypreny-PP
2.5.1.88
2.5.1.68
(Z,E)-Farnesyl-PP
Diterpenoid biosynthesis
Carotenoid biosynthesis
Indole diterpene alkaloid biosynthesis
Farnesal
1.1.1.216
1.1.1.354
trans-Farnesol
2.7.1.216
(E,E)-Farnesyl-P
2.7.4.32
2.5.1.10
(E,E)-Farnesyl-PP
3.1.7.6
1.8.3.5
1.8.3.6
Farnesyl cysteine
Geranyl geranyl-PP
Steroid biosynthesis
2.5.1.29
PCME
2.5.1.82
2.5.1.89
1.3.1.83
2.5.1.85
2.5.1.84
3.4.24.84
FACE2
2.1.1.100
2.5.1.58
2.5.1.83
Protein C-terminal S-farnesyl-L-cysteine methyl ester
Protein C-terminal S-farnesyl-L-cysteine
S-farnesyl protein
Hexaprenyl-PP
tritrans,heptacis-Undecaprenyl-PP
Phytyl-PP
Nona-prenyl-PP
Carotenoid biosynthesis
2.5.1.30
Heptaprenyl-PP
N-Glycan biosynthesis
Dolichol-PP
Dehydro dolichol-PP
2.5.1.87
2.5.1.90
Octaprenyl-PP
2.5.1.31
di-trans,poly-cis-Undecaprenyl-PP
2.5.1.91
Decaprenyl-PP
Ubiquinone and other terpenoid-quinone biosynthesis
00900 6/26/18
(c) Kanehisa Laboratories

Figure S6

References

Anderson, A. J., and Dawes, E. A. (1990). Occurrence, metabolism, metabolic role, and industrial uses of bacterial polyhydroxyalkanoates. *Microbiol. Rev.* 54, 450–472. doi:10.1016/0378-1097(92)90313-D.

Berry, A., Janssens, D., Hümbelin, M., Jore, J. P. M., Hoste, B., Cleenwerck, I., et al. (2003). *Paracoccus zeaxanthinifaciens* sp. nov., a zeaxanthin-producing bacterium. *Int. J. Syst. Evol. Microbiol.* 53, 231–238. doi:10.1099/ijs.0.02368-0.

Cantera, S., Lebrero, R., Sadornil, L., García-encina, P. A., and Munoz, R. (2016). Valorization of CH_4 emissions into high-added-value products : Assessing the production of ectoine coupled with CH_4 abatement. *J. Environ. Manage.* 182, 160–165. doi:10.1016/j.jenvman.2016.07.064.

Celińska, E., and Grajek, W. (2009). Biotechnological production of 2,3-butanediol-Current state and prospects. *Biotechnol. Adv.* 27, 715–725. doi:10.1016/j.biotechadv.2009.05.002.

Levett, I., Birkett, G., Davies, N., Bell, A., Langford, A., Laycock, B., et al. (2016). Techno-economic assessment of poly-3-hydroxybutyrate (PHB) production from methane - The case for thermophilic bioprocessing. *J. Environ. Chem. Eng.* 4, 3724–3733. doi:10.1016/j.jece.2016.07.033.

Xiao, Z., and Lu, J. R. (2014). Strategies for enhancing fermentative production of acetoin : A review. *Biotechnol. Adv.* 32, 492–503. doi:10.1016/j.biotechadv.2014.01.002.

Zhang, C. (2018). Biosynthesis of carotenoids and apocarotenoids by microorganisms and their industrial potential. *IntechOpen*. doi:10.5772/intechopen.79061.
